# Using Ultrasound-Treated Washout from Conifer Needles and Fresh Snow Samples in Air Pollution Monitoring

**DOI:** 10.1155/2020/3529437

**Published:** 2020-06-23

**Authors:** A. Kholodov, M. Tretyakova, K. Golokhvast

**Affiliations:** ^1^Far East Geological Institute, Far East Branch of Russian Academy of Sciences, Vladivostok, Russia; ^2^Far Eastern Federal University, Vladivostok, Russia; ^3^N.I. Vavilov All-Russian Institute of Plant Genetic Resources, Saint-Petersburgh, Russia; ^4^Pacific Geographical Institute, Far Eastern Branch of Russian Academy of Sciences, Vladivostok, Russia

## Abstract

Snow precipitation and snowpack are commonly used to assess the condition of the aerial environment. Another way to monitor air quality is to study trees and shrubs, which are natural barriers for capturing air pollution, including atmospheric particulate matter. The hypothesis of the current study was that using fresh snow precipitation and washout from vegetation for the monitoring of air pollution can produce comparable results. In this study, we compared the results of laser diffraction analysis of suspended particular matter in melted fresh snow and ultrasound-treated washout from conifer needles. The samples were collected at several sites in Primorsky Krai, Russian Federation, and analyzed according to the same scheme. We observed that the content of particulate matter with a smaller aerodynamic diameter in the ultrasound-treated washout from conifer needles was higher than that in the melted fresh snow. The content of PM_10_ in the ultrasound-treated washout from conifers was increased by 6–27% depending on the site, showing greater efficacy of this method. This method can be used as an alternative to the sampling of snow for the monitoring of ambient air pollution, taking into account several limitations.

## 1. Introduction

Snow precipitation and snowpack are commonly used to assess the condition of the aerial environment in urban areas and at remote sites [1, 2]. Falling snow scavenges air pollutants of both anthropogenic and natural origins [3, 4]. Due to their larger surface area and slower deposition speed, snowflakes absorb more air pollutants compared to raindrops [5]. Urban snowpack is further polluted by the products of combustion, motor vehicle emissions, winter road maintenance chemicals, debris, and litter, turning it into a “toxic cocktail of pollutants” [6, 7]. For certain types of analysis, e.g., for particle size distribution analysis of melted fresh snow, the sample preparation is minimal. Also, snow samples are inexpensive and easy to collect and handle.

However, snow sampling has its limitations. The main limitation is seasonality, meaning that scientists need to switch to other sampling methods during the snowless period. The time of sampling is also important. Snowpack samples must be collected before melting, and rain-on-snow events can destroy the samples [8]. Fresh snow should be collected during or immediately after the snowfall.

Another way to monitor air quality is to study the vegetation. Trees and shrubs are natural barriers for capturing air pollution, including atmospheric particulate matter (PM) [9]. Due to their leaf structure and other properties, conifers are more effective in accumulating fine and coarse particles (aerodynamic diameter (Dp) > 1 *μ*m), while broad-leaved species accumulate more ultrafine particles (Dp < 1 *μ*m) [10]. PM_10_ particles are a complex heterogeneous mixture of solids and liquid droplets floating in the air in the size range of 2.5–10 *μ*m in diameter and, depending on the composition, they may pose a threat to human health [11].

A comprehensive study by Nowak et al. [9] reported that urban trees remove about 215 thousand tons of PM_10_ in the US per year. A study at two sites in Europe (Norway and Poland) ranked 47 woody species by their ability to accumulate roadside PM, and it was found that *Pinus sylvestris* was among the species most efficient in capturing PM [12]. Another experimental study in Beijing Botanical Garden showed that two pine species, *Pinus tabuliformis* and *Pinus bungean*, captured more total suspended particles per unit leaf area compared to broad‐leaved tree species [13].

The goal of this study was to verify if using fresh snow and vegetation for environmental monitoring could produce comparable results. For this purpose, we compared the results of particle size distribution analysis of suspended particular matter in melted fresh snow and ultrasound-treated washout from conifer needles. The samples were collected at several sites and analyzed according to the same scheme.

## 2. Experiments

In this study, we compared the results of the particle size analysis of melted snow and ultrasound-treated washout from conifer needle samples collected at several sites in Primorsky Krai, Russian Federation. Conifer species used for this study were Khingan fir (*Abies nephrolepis*), Manchurian fir (*Abies holophylla*), Korean pine (*Pinus koraiensis*), and Scots pine (*Pinus sylvestris*).

  The first site (*n*_1_ = 3, *n*_2_ = 3, where *n*_1_ is the number of snow samples at the site and *n*_2_ is the number of conifer needle samples at the site) is a small urban settlement Terney (45°02' N, 136°36' *E*; area of 3.5 km^2^, >3000 inhabitants) located in an environmentally favorable area near the Sikhote-Alin nature reserve. The samples were collected in the winter of 2017. The data used in this study are modified from previous research by Kodintsev et al. [14].
  The second site (*n*_1_ = 8, *n*_2_ = 10) is the Spassk–Dalny town (44°36' N, 132°49' *E*; area of 43.5 km^2^, >40,000 inhabitants). The cement plant located in the town is a recognized source of environmental pollution. The snow samples were collected in the winter of 2017–2018, and the confer needle samples were collected in the summer of 2018.
  The third site (*n*_1_ = 8, *n*_2_ = 10) is the Nakhodka city (42°49' N, 132°52' *E*; area of 325.9 km^2^, >147,000 inhabitants), mainly its port complex, which is the second largest port in Russia in terms of cargo turnover. The port complex currently deals with the transshipment of coal. The samples were collected in January of 2020.

Snow samples were collected from 1 m^2^ area into 3-liter plastic containers prewashed with distilled water. To avoid the secondary pollution of settled snow, only the top layer of fresh snow was collected. After collection, the snow samples were transported to the laboratory of Research and Educational Center Nanotechnology (FEFU), where the melted snow was analyzed for particle size distribution.

Conifer needles were collected according to the previously described method [14, 15] from trees at the height of 1–1.5 m and carefully transported to the lab. In the laboratory, the containers with sample needles were filled with double-distilled water and cleaned with ultrasound using a Sonopulse 3100 HD ultrasonic homogenizer (Bandelin electronic GmbH & Co. KG, Germany) at 22 kHz, 100 watts, and a 5-minute exposure to remove the pollution particles from the needles. The settings were determined after a series of experiments, where the surface of needles was examined using a microscope after ultrasound cleaning at various settings to verify that most of the fine dust particles were cleaned from the surface into the solution. No additional preparation of conifer needle samples was made.

Both the melted snow samples and the ultrasound-treated washout from conifer needle samples were analyzed for particle size distribution using the Analysette 22 NanoTec plus laser particle sizer (Fritsch GmbH, Germany). The measurements were run at the settings of quartz/water at 20°C in three repeats. The results were analyzed with Fritch MaS software using the Mie–Gruneisen equation of state. Statistical analyses were performed in the software package STATISTICA 10 (StatSoft, Inc., USA).

## 3. Results

The suspended particles were classified into seven groups depending on their aerodynamic diameter: (1) 0.1–1 *µ*m (PM_1_), (2) 1–10 *µ*m (PM_10_), (3) 10–50 *µ*m, (4) 50–100 *µ*m, (5) 100–400 *µ*m, (6) 400–700 *µ*m, and (7) ≥ 700 *µ*m [16]. This classification was used in the interpretation of the laser particle size analysis results.

**Figure 1:**
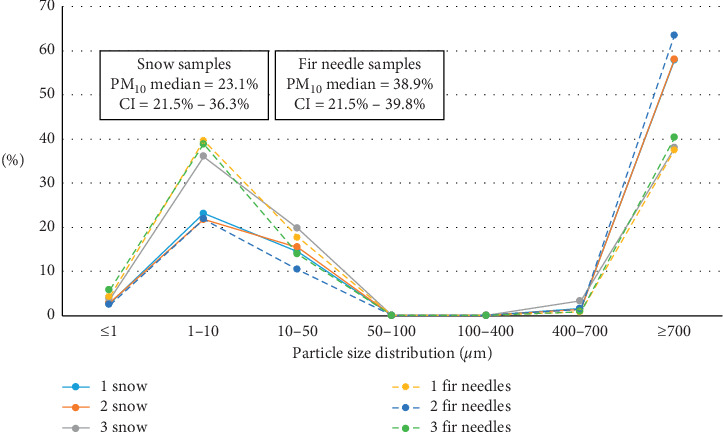
Particle size distribution of particulate matter measured in melted snow samples and in ultrasound-treated washout from conifer needle samples from Terney town. The figure is based on the data from [14].

**Figure 2:**
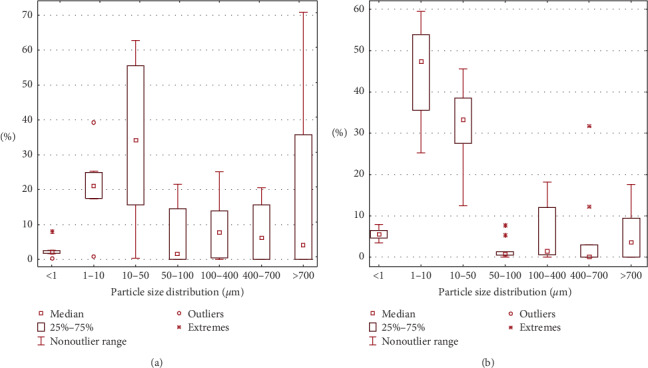
Statistical data of PM particle size distribution in Spassk–Dalny town (a) in melted snow samples and (b) in ultrasound-treated washout from conifer needle samples.

**Figure 3:**
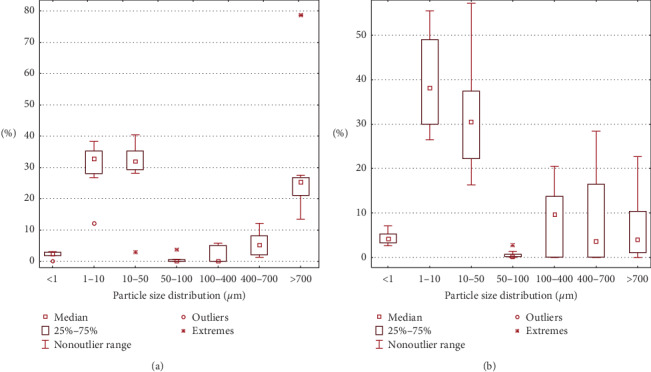
Statistical data of PM particle size distribution in Nakhodka city; (a) in melted snow samples and (b) in ultrasound-treated washout from conifer needle samples.

The particle size distribution patterns in the samples from the first site (Terney settlement, fresh snow, and conifer needles) are closely comparable (Figure 1). The median of the content of PM_10_ in the washout from conifer needle samples is higher by >15%. It should be mentioned here that the number of samples collected at this pilot site did not allow us to present significant graphs of statistical data, unlike other sites used in this study (Figures 2 and 3).

**Figure 4:**
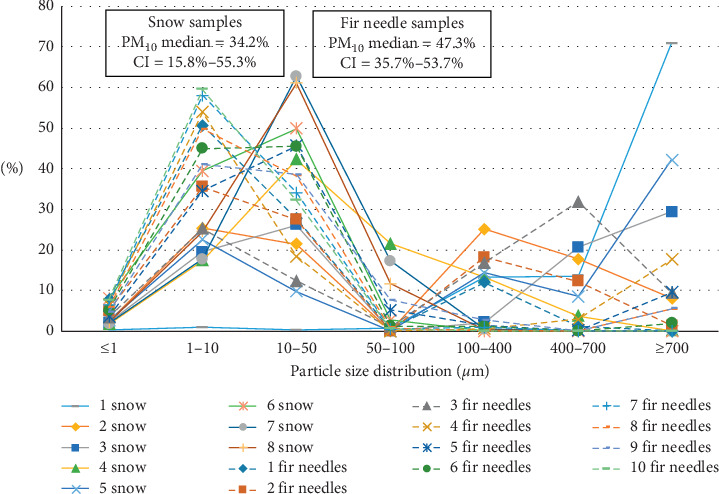
Particle size distribution of particulate matter measured in melted snow samples and in ultrasound-treated washout from conifer needle samples from Spassk–Dalny town.

The second site (Spassk–Dalny) is an industrial town with a cement plant. The industrial impact on the atmosphere manifests in the increased content of smaller fractions of particulates in the washout from conifer needle samples. In the melted snow, the suspended particles are distributed more evenly (PM_10_ median is 20.9%), while in the conifer needles, there is an increase in the content of both PM_1_ and PM_10_ (PM_10_ median is 47.3%) (Figures 2 and 4).

**Figure 5:**
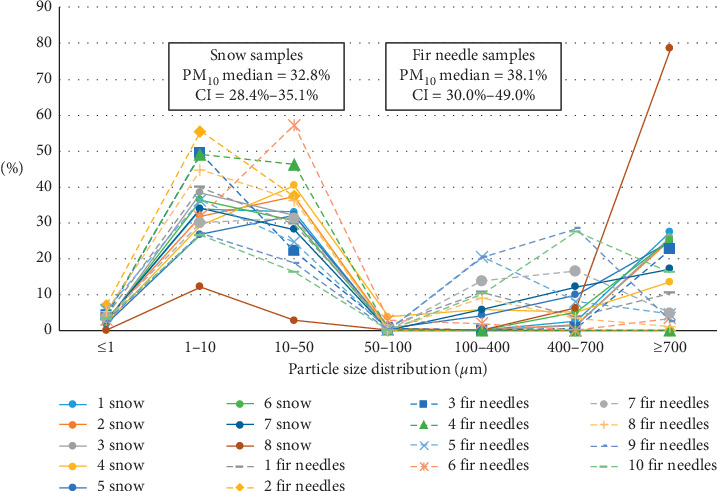
Particle size distribution of particulate matter measured in melted snow samples and in ultrasound-treated washout from conifer needle samples from Nakhodka city.

The samples from Nakhodka city demonstrate the same trend: the average content of finer fractions is higher in conifer needle samples. The content of PM_1_ is almost twice high (2.3% and 4.2%), and PM_10_ content is increased by 5.3% (PM_10_ median in snow samples is 32.8%, in conifer needles 38.1%). The PM_10_ values are relatively high and reflect the industrial nature of the particulate matter in the sampling area (Figures 3 and 5).

The increased content of smaller fractions in an ultrasound-treated washout from conifer needle samples observed in the study may be caused by the fact that due to their surface properties, conifer needles capture more PM. Another reason may be that the ultrasound treatment breaks particle aggregates into PM of smaller aerodynamic diameter.

## 4. Discussion

In the study, we collected freshly fallen snow to assess the pollutants that were in the air, without the burden of secondary pollution in the snow cover. The range and the extent of pollutants in snow cover are known to be higher than that in the precipitation [17]. Even more so, the pollutants may react with snow, and resulting admixtures are released into melt water and soil during winter even in cold climate due to temperature fluctuations and thaws [6]. Due to this, even snowpack does not accurately represent the accumulated pollution.

Our study showed a higher content of PM fractions with a smaller aerodynamic diameter in the washout from conifer needles compared to the fresh snow. This trend was observed in all three sites: 15.8% increase in site 1 (Terney town), 13.1% in site 2 (Spassk–Dalny town), and over 5.3% in site 3 (Nakhodka city). One of the reasons for this trend is that conifer tree species are effective in capturing fine atmospheric PM on their surface [9, 11, 13].

Another reason for the elevated content of smaller PM in conifer needles may be the use of an ultrasound cleaning technique. Washing the sticky leaf-needles efficiently to get the pollutants into the solution was a challenge in the study. Hand washing and shaking of sample material still left pollutant particles on the surface of samples [18]. A known way to clean the surface of plant samples is ultrasonic treatment. In 1979, Godzik et al. [19] used chloroform and ultrasonic cleaning technique to remove particulates from the leaves of oak and pine. The method was modified (chloroform was substituted with water) and further used to remove pollutants from various types of plants, vegetation, and fresh produce [20–22]. Both ultrasonic probes and baths are used with low frequencies ranging from 20 to 40 kHz. Experimentally, we found that the parameters of 22 kHz, 100 watts, and a 5-minute exposure removed most particles from leaves and needles. The increase of PM_10_ particles in the solution was by 10.4%–26.6% compared to simply rinsing the conifer needles in the water [23]. While this is an effective method to get particulates into the solution, it has its limitations, as it was reported that ultrasonic treatment may cause a decrease in particle size and narrow the distribution of particulates [24].

In earlier studies of the authors of this article/paper, the dried sediment from melted snow and ultrasound-treated washout from conifer needles was effectively used for further analysis of the environment. The morphology of particles, the content of heavy metals, and Raman spectra of particles were analyzed, allowing them to identify metals, cement, and coal in ambient PM [15, 25, 26].

A sampling of snow and conifer needles makes it possible to conduct inexpensive environmental monitoring regardless of the season and get comparable results. However, certain limitations of both methods should be taken into consideration. In territories with colder climate, anthropogenic emissions of potentially toxic substances from coal combustion are much higher in the heating season than during the nonheating season. While coniferous species are efficient in the removal of particulate matter from ambient air during the heating season [27], they are exposed to contamination for long time periods and that may affect the monitoring results. In the snowless season, rainstorms should be taken into account, which can naturally wash the surface of leaves and clean them from pollutants [28, 29].

## 5. Conclusions

In the study, we compared the results of laser diffraction analysis of melted fresh snow samples and ultrasound-treated washout from conifer needle samples taken at three sites. Our results showed greater efficacy of ultrasound washout from conifer needles in the study of suspended particles, as this method allows a greater number of smaller particles to be deposited into the solution. This method can be used as an all-season alternative to the sampling of snow for the monitoring of ambient air pollution. Certain limitations of both methods should be considered.

The assessment of air quality is an important part of environmental monitoring. The sampling and analysis methods described in this study are relatively easy and cost-effective ways of monitoring, especially in conditions of remote areas and smaller settlements.

## Data Availability

The data used to support the findings of this study are available from the corresponding author upon request.
